# Late clinical failure associated with cytochrome b codon 268 mutation during treatment of falciparum malaria with atovaquone–proguanil in traveller returning from Congo

**DOI:** 10.1186/s12936-020-3126-y

**Published:** 2020-01-21

**Authors:** Laurencie Massamba, Marylin Madamet, Nicolas Benoit, Alicia Chevalier, Isabelle Fonta, Véronique Mondain, Pierre-Yves Jeandel, Rémy Amalvict, Pascal Delaunay, Joel Mosnier, Pierre Marty, Christelle Pomares, Bruno Pradines

**Affiliations:** 1 Parasitologie Mycologie, Centre Hospitalo-Universitaire de Nice, Université de la Côte d’Azur, Nice, France; 2 Unité Parasitologie et entomologie, Département de Microbiologie et de maladies infectieuses, Institut de recherche biomédicale des armées, Marseille, France; 3 Aix Marseille Université, IRD, SSA, AP-HM, VITROME, Marseille, France; 4 IHU Méditerranée Infection, Marseille, France; 5 Centre national de référence du Paludisme, Marseille, France; 6 Infectiologie, Centre Hospitalo-Universitaire de Nice, Université de la Côte d’Azur, Nice, France; 7 Service de Médecine Interne, Centre Hospitalier Universitaire de Nice, Nice, France; 8 MIVEGEC, UMR IRD 224-CNRS 5290-Université de Montpellier, Montpellier, France; 9 INSERM, U1065, Centre Méditerranéen de Médecine Moléculaire, Faculté de Médecine, Virulence microbienne et signalisation inflammatoire, Nice, France

**Keywords:** Malaria, *Plasmodium falciparum*, Anti-malarial drug, Resistance, In vitro, Atovaquone, Proguanil, Cytochrome b

## Abstract

**Background:**

The drug combination atovaquone–proguanil, is recommended for treatment of uncomplicated falciparum malaria in France. Despite high efficacy, atovaquone–proguanil treatment failures have been reported. Resistance to cycloguanil, the active metabolite of proguanil, is conferred by multiple mutations in the *Plasmodium falciparum* dihydrofolate reductase (*pfdhfr*) and resistance to atovaquone by single mutation on codon 268 of the cytochrome b gene (*pfcytb*).

**Case presentation:**

A 47-year-old female, native from Congo and resident in France, was admitted in hospital for uncomplicated falciparum malaria with parasitaemia of 0.5%, after travelling in Congo (Brazzaville and Pointe Noire). She was treated with atovaquone–proguanil (250 mg/100 mg) 4 tablets daily for 3 consecutive days. On day 5 after admission she was released home. However, many weeks after this episode, without having left France, she again experienced fever and intense weakness. On day 39 after the beginning of treatment, she consulted for fever, arthralgia, myalgia, photophobia, and blurred vision. She was hospitalized for uncomplicated falciparum malaria with a parasitaemia of 0.375% and treated effectively by piperaquine–artenimol (320 mg/40 mg) 3 tablets daily for 3 consecutive days. Resistance to atovaquone–proguanil was suspected. The Y268C mutation was detected in all of the isolates tested (D39, D42, D47). The genotyping of the *pfdhfr* gene showed a triple mutation (N51I, C59R, S108N) involved in cycloguanil resistance.

**Conclusion:**

This is the first observation of a late clinical failure of atovaquone–proguanil treatment of *P. falciparum* uncomplicated malaria associated with *pfcytb* 268 mutation in a traveller returning from Congo. These data confirm that the Y268C mutation is associated with delayed recrudescence 4 weeks or more after initial treatment. Although atovaquone–proguanil treatment failures remain rare, an increased surveillance is required. It is essential to declare and publish all well-documented cases of treatment failures because it is the only way to evaluate the level of resistance to atovaquone.

## Background

The drug combination atovaquone–proguanil, trade name Malarone, is recommended as the second-line treatment of uncomplicated falciparum malaria in adults in France [1]. Despite high efficacy, atovaquone–proguanil treatment failures have been reported [2–16]. Resistance to cycloguanil, the active metabolite of proguanil, is conferred by multiple mutations in the *Plasmodium falciparum* dihydrofolate reductase (*pfdhfr*) (N51I, C59R and S108N) [17]. Resistance to atovaquone is conferred by single mutation Y268S on the cytochrome b gene (*pfcytb*) [2–4, 8–11, 13], and much more rarely Y268C [3, 5, 7, 16] and Y268N [14]. In addition, resistance to atovaquone was also reported in patients with parasites without codon 268 mutation [5, 6, 13, 15]. Parasites carrying *pfcytb* codon 268 mutations are associated with delayed recrudescence 4 weeks or more after initial treatment, whereas recrudescence of parasites without codon 268 mutation appears more precociously after initial treatment [5, 18]. However, well-documented atovaquone–proguanil treatment failure remains extremely rare. This is the first report of genetic confirmation of atovaquone–proguanil resistance in *P. falciparum* isolate acquired in Congo.

## Case presentation

A 47-year-old female, native from Congo and resident in France, was admitted 15 June, 2019 to the Emergency Unit of a private hospital in Nice, France. She had presented fever, headache and abnormal weakness for 2 days prior to admission. She had no history of underlying diseases, but had recently travelled to Congo (Brazzaville and Pointe Noire) from 31 May to 13 June, 2019. During her stay she took halofantrine as anti-malarial prophylaxis medication. This anti-malarial drug is not recommended for malaria prophylaxis. Halofantrine presents a risk of cardiac toxicity and its absorption is unreliable. On admission, her physical examination revealed fever, headache, arthralgia, and myalgia. No neurological deficits were found and the patient was haemodynamically stable. Laboratory studies showed C reactive protein of 62 mg/L, a haemoglobin concentration of 10.2 g/dL, a haematocrit 34%, a mean corpuscular volume (MCV) of 76 fL and a white cell count 3190 cells/mm^3^ with 79.3% neutrophils, 12.9% lymphocytes and 0.6% eosinophils. The platelet count was 159,000 cells/mm^3^. The liver function showed a hepatic cytolysis (ASAT 151 U/L; ALAT 129 U/L). The peripheral blood smear revealed the presence of trophozoïtes of *P. falciparum* with 0.5% of parasitaemia. She weighed 90 kg. She was hospitalized and treated on 16 June, 2019 at 1h30 am by atovaquone–proguanil (250 mg/100 mg) (Malarone^®^) 4 tablets daily for 3 consecutive days. The drug intake with food was monitored by nurses. The patient experienced no vomiting or diarrhea after drug administration. The patient was apyretic the day after the first dose of atovaquone–malarone. Control of parasitaemia, performed 3 days after the beginning of treatment, was 0.1%. On day 5 after admission, she did not show complaints or signs of any disease and parasites and was released home. However, many weeks after this episode, without any subsequent travel, she again experienced fever and intense weakness. On day 39 (24 July) after the beginning of treatment and because of the aggravation of symptoms, she consulted for fever, arthralgia, myalgia, photophobia, and blurred vision at the Emergency Unit of the teaching hospital of Nice. A blood smear stained with Giemsa revealed the presence of trophozoïtes of *P. falciparum* with 0.375% of parasitaemia. Resistance to atovaquone–proguanil was suspected. She was hospitalized in the Internal Medicine unit where she received medication of piperaquine-artenimol (320 mg/40 mg) 3 tablets daily for 3 consecutive days. After initiation of treatment the patient’s clinical outcome improved: fever, headache and myalgia disappeared and only weakness remained and lasted for several weeks after her stay in the teaching hospital. Peripheral blood smear controls performed at day 42, day 49 and day 66 (day 3, 7 and 27 after the second cure of treatment, respectively) found no trophozoïte and the patient was considered cured from her malaria episode.

The blood sampled for malaria researches were sent to the French National Reference Centre for Imported Malaria Study Group of Marseille (France) where Sanger sequencing of *pfctyb* for atovaquone resistance, *pfdhfr* for cycloguanil resistance, *P. falciparum* chloroquine resistance gene (*pfcrt*) for chloroquine resistance, *P. falciparum* Kelch propeller gene (*K13*) for artemisinin resistance and *P. falciparum* multidrug resistance 1 gene (*pfmdr1*) for lumefantrine resistance was performed as previously described [19–21]. The Y268C mutation was detected in all of the isolates collected during clinical treatment failure and follow-up (D39, D42, D47). The pre-treatment (D0) was not available. The molecular data are presented in Table 1. The genotyping of the *pfdhfr* gene showed a triple mutation (N51I, C59R, S108N) involved in cycloguanil resistance. The haplotype CVIET, associated with chloroquine resistance, was found [22]. No mutation was found in K13 propeller domain. The parasites carried a wild haplotype NYSND (codons 86, 184, 1034, 1042, 1246 on *pfmdr1*).

**Table 1:** Single nucleotide polymorphisms identified in *P. falciparum* cytochrome b gene (*pfcytb*), *P. falciparum* dihydrofolate reductase gene (*pfdhfr*), *P. falciparum* chloroquine resistance transporter gene (*pfcrt*), *P. falciparum* multidrug resistance 1 gene (*pfmdr1*), *P. falciparum* kelch propeller gene (*pfK13*) of samples collected at day 39, 42 and 47

Collection day	*pfcytb*	*pfdhfr*				*pfcrt*					*pfmdr1*					*pfK13*
39	268C	51I	59R	108 N	I164	C72	73 V	74I	75E	76T	N86	Y184	S1034	N1242	D1246	WT
42	268C	51I	59R	108 N	I164	C72	73 V	74I	75E	76T	N86	Y184	S1034	N1242	D1246	WT
47	268C	51I	59R	108 N	I164	C72	73V	74I	75E	76T	N86	Y184	S1034	N1242	D1246	WT

*WT* wild type

## Discussion and conclusion

This is the first described observation of a late clinical failure of atovaquone–proguanil treatment of *P. falciparum* uncomplicated malaria associated with *pfcytb* 268 mutation in a traveller returning from Congo. The Y268C mutation was identified in recrudescence on day 39 after initial treatment by atovaquone–proguanil. These data confirm analyses that showed that *pfcytb* codon 268 mutations are associated with delayed recrudescence 4 weeks or more after initial treatment [5, 18]. The cases of early treatment failures are not associated with codon 268 mutation [5, 6, 13, 15]. Although atovaquone–proguanil treatment failures remain rare, increased surveillance is required. It is essential to declare and publish all well-documented cases of treatment failures because it is the only way to evaluate the level of resistance to atovaquone. It is difficult to monitor atovaquone resistance by using in vitro testing or *pfcytb* mutation detection in general surveys in local and global parasites or in pre-treatment isolates. The codon 268 mutation or in vitro decreased susceptibility are rarely found in initial *P. falciparum* parasites before atovaquone–proguanil treatment and clinical failure [2, 4, 5, 8–16, 23] and in general surveys on unexposed *P. falciparum* parasites to atovaquone–proguanil due to low selective pressure in endemic areas [4, 19, 24–29]. As no D0 sample was available, it is difficult to conclude on whether this resistance was acquired during atovaquone–proguanil treatment or transmitted. However, this resistance is in almost all the cases described associated with acquisition and selection of cytochrome b mutation by parasites already resistant to cycloguanil during atovaquone–proguanil treatment [2–4, 10, 13, 16, 30]. Another hypothesis which argues for acquired resistance during atovaquone–proguanil treatment is that parasites carrying the 268C mutation in *P. berghe*i ANKA *cyb* gene are unable to produce sporozoïte stages in the mosquito salivary glands or to infect mouse [31]. These parasites successfully generated oocysts but these oocysts had developmental defects. This lack of transmission also explains the low prevalence of *pfcytb* 268 mutations in endemic areas and the spread of atovaquone–proguanil resistance.

Plasma drug concentration is a factor of treatment failure. In absence of atovaquone plasma concentration measurement, underdosing seems anyway to be ruled out. Atovaquone–proguanil was correctly administered with food and the intake was monitored by nurses. The patient experienced no vomiting or diarrhea after drug administration. She weighed 90 kg. Patients with a body weight > 100 kg have a marked increased chance of treatment failure by underdosing compared with < 100 kg [32].

The investigation of atovaquone–proguanil treatment failure should continue and be reinforced in order to identify emergence and to monitor the spread of atovaquone resistance, and even more, if atovaquone–proguanil would be associated with artesunate or not as an alternative to artemisinin combination therapy in areas where *P. falciparum* parasites are multi-drug resistant, such as in Cambodia [29, 33].

## Data Availability

The datasets analysed in this study are available from the corresponding author on reasonable request.

## Abbreviations

ALAT: alanine aminotransferase
ASAT: aspartate aminotransferase
MCV: mean corpuscular volume
*pfcrt*: *P. falciparum* chloroquine resistance gene
*pfcytb*: *P. falciparum* cytochrome b gene
*pfdhfr*: *P. falciparum* dihydrofolate reductase
*K13*: *P. falciparum* Kelch propeller gene
*pfmdr1*: *P. falciparum* multidrug resistance 1 gene
